# The Correlation of Decreased Heart Rate Recovery and Chronotropic Incompetence with Exercise Capacity in Idiopathic Pulmonary Arterial Hypertension Patients

**DOI:** 10.1155/2017/3415401

**Published:** 2017-02-12

**Authors:** Changwei Wu, Jian Guo, Hui Liu, Bigyan Pudasaini, Wenlan Yang, Qinhua Zhao, Lan Wang, Jinming Liu

**Affiliations:** ^1^Department of Pulmonary Function Test, Shanghai Pulmonary Hospital, Tongji University School of Medicine, Shanghai 200433, China; ^2^Department of Respiratory and Critical Illness, Henan Provincial Peoples' Hospital of Zhengzhou University, Henan 450003, China; ^3^Department of Pulmonary Circulation, Shanghai Pulmonary Hospital, Tongji University School of Medicine, Shanghai 200433, China

## Abstract

We show by this study that a decrease in HRR1 in IPAH patients is associated with severe limitation of exercise capacity. HRR1 < 16 beats and CI just after completion of a CPET could be an indicator of poor prognosis.

## 1. Introduction

Pulmonary arterial hypertension (PAH) is a rapidly progressive pulmonary vascular disease characterized by pulmonary vascular growth that leads to increased pulmonary vascular pressure and right-sided heart failure, consequently leading to impaired exercise capacity and death [1, 2]. It remains a complex disease with many challenges, including late diagnosis, suboptimal treatment adherence, variability in clinical trial designs, and limited morbidity, mortality, and economic outcomes data. Research and programs that focus on improvements in these areas will provide relevant information to assist healthcare providers and funding agencies in their decision making process [3, 4].

Peak VO_2_ in CPET is a widely used variable and has proved to be useful in diagnosing and evaluating IPAH patients [5, 6]. However, unlike peak VO_2_, the decline of heart rate recovery after exercise does not require maximal effort. It has been confirmed to be promising and an available method in assessing functional impairment in chronic heart failure (CHF) patients [7, 8]. Chronotropic incompetence (CRI) and low heart rate recovery (HRR) have been assumed to be an index of autonomic imbalance [9]. Earlier studies have proved that nervous system dysfunction is frequent in PAH patients and heart rate recovery at one minute (HRR1) <16 is a predictor of clinical deterioration [10]. However, no previous study has assessed the relation between HRR1 in CPET and exercise capacity in a relatively large IPAH cohort. Therefore, we retrospectively investigated CPET data along with other clinical indices in fifty-eight IPAH patients and contrast HRR1 and CR with twenty-five age and gender matched controls. Furthermore, based on the heart rate recovery the patients with IPAH were divided into normal heart rate recovery group (*n* = 27) and abnormal group (*n* = 31). We hypothesized that lower HRR1 or CR would be associated with a worse exercise capacity.

## 2. Methods

### 2.1. Patients and Control Subjects

Fifty-eight patients with established IPAH based on currently accepted diagnostic criteria (Nice, 2013) and clinical and laboratory data, including right heart catheterization (RHC), were included. CPET and pulmonary function test (PFT) data of 25 healthy subjects who were free of cardiorespiratory and metabolic diseases based on standard clinical functional (CPET and PFT) and laboratorial evaluations were also included. All of the participants that were involved in the study signed written informed consent. This study was approved by the Institutional Ethics Committee at Shanghai Pulmonary Hospital. Those that did not meet the standard recommended diagnostic criteria for IPAH were excluded from the study [9]. The data included only the first PFT, CPET, and N-terminal pro-brain nitric peptide (NT-proBNP) measurements made after referral to our hospital, almost always prior to the initiation of pulmonary vasodilator therapy. We made sure that no IPAH patients included were using drugs that could slow heart rate, such as beta-blockers. But all of them were under targeted drug therapy.

### 2.2. Right Heart Catheterization

All IPAH patients underwent RHC. Mean hemodynamic measurements included mean pulmonary arterial pressure (mPAP), pulmonary artery wedge pressure (PAWP), and mean right atrium pressure (mRAP). Cardiac output (CO) was obtained using the thermodilution method. Pulmonary vascular resistance (PVR) was calculated using standard formulas: PVR = (mPAP − PAWP)/CO.

### 2.3. Six-Minute Walking Test

The test was performed on duplicate in a 100 m-long corridor following the guidelines of the American Thoracic Society [10]. Six-minute walking distance (6MWD; in meters) was the largest recorded value.

### 2.4. PFT Measurements

Each patient and normal subject underwent resting measurements of forced vital capacity (FVC), forced expiratory volume in 1 sec (FEV1), diffusing capacity of carbon monoxide (DLco), forced expiratory flow at 25% of forced vital capacity (FEF25), forced expiratory flow at 50% of forced vital capacity (FEF50), and forced expiratory flow at 75% of forced vital capacity (FEF75) using ATS/ERS criteria and their results were reported in absolute terms and % predicted values (e.g., FVC% and PEF50%) [11–13].

### 2.5. CPET Procedure and Data Collection

After familiarization with the exercise apparatus, each patient performed a symptom-limited incremental cardiopulmonary exercise testing (CPET; 5–15 W/min for patients with IPAH and 20–25 W/min for the controls) to maximal tolerance on an electromagnetically braked cycle ergometer (Masterscreen-CPX, Jaeger, Hoechberg, Germany) as part of their initial evaluation at our center, which is a referral center for pulmonary vascular diseases. Before each test, the equipment was calibrated according to the manufacturer's specifications using reference and calibration gases. Standard 12-lead electrocardiograms (ECGs) and pulse oximetry were continuously monitored. Systemic blood pressure was measured every two minutes and at exercise cessation by an automatic cuff. The exercise protocol consisted of 3 min of rest and 3 min of unloaded cycling at 60 revolutions per minute. The rate of increasing work depended on the estimated exercise capacity of the subjects. Subjects were encouraged to exercise to the limits of their functional capacities or until the physician stopped the test because of severe adverse events, such as chest pain, light-headedness, potentially life-threatening arrhythmias, ST-segment changes, or marked systolic hypotension. Most CPET values were reported in absolute terms and normalized to percentage of predicted (% pred). Predicted values were calculated using accepted equations [14].

### 2.6. CPET Data Calculations

Carbon dioxide output (VCO_2_, ml/min, STPD), VO_2_ (ml/min, STPD), VE (l/min, BTPS), and tidal volume (l, BTPS) were measured continuously breath by breath using a CPX Metabolic Measurement Cart (Masterscreen-CPX, Jaeger, Hoechberg, Germany) that was equipped with rapidly responding O_2_ and CO_2_ analyzers. Data were recorded as mean of 10 sec. Peak VO_2_ was defined as the highest 30 sec average of VO_2_, and other peak parameters were calculated at the same time. Each AT was determined by the V-slope method [15]. Heart rate was obtained from the R-R distance as established by an in-built 12-lead electrocardiogram. VE-VCO_2_ slope was determined by linear regression analysis of the relation between VE and VCO_2_ during exercise, excluding data above the ventilatory compensation point [16].

Heart rate recovery at 1 minute (HRR1) was defined as the difference between peak HR and HR registered after 1 minute of active recovery. CRI was diagnosed if there was a CR failure to reach 0.8 (chronotropic reserve [CR, %] = [peak HR − resting HR/220 – age − resting HR] × 100). After Sun et al., we defined HRR1 < 16 as a poor prognosis in IPAH patients.

### 2.7. Statistical Analysis

Microsoft Office-2007, SPSS-19.0, and Origin-8.0 computer software were used. Data are expressed as mean ± SD, except where specifically noted. Most PFT and CPET values are expressed in absolute terms and % pred. *P* < 0.05 was considered significant. We used the *t*-test and *χ*^2^ test for data comparison between the two groups. Correlations between HRR1 and other variables were determined by Pearson's correlation test, except for New York Heart Association functional classification (NYHA FC) by Spearman rank correlation test.

## 3. Results

### 3.1. Baseline Clinical and Demographic Characteristics

PFT and CPET parameters of the healthy group were within normal limits. The FEV1/FVC, DL_CO_, and other PFT values were significantly lower in IPAH patients as compared with normal subjects. Characteristics of patients and healthy subjects are detailed in Table 1.

All subjects completed their CPET without incident. Almost all patients stopped exercise because of fatigue and/or acute shortness of breath. Rarely, patients noted palpitations or light-headedness and recovered after resting for few minutes. All subjects declared they had done their best.

The magnitude of the absolute and percentage of all CPET parameters of oxygen uptake were strikingly abnormal in IPAH patients. The HRR1 and CR values were significantly lower in IPAH patients compared with the normal. The incidence of CRI in IPAH (89.7%) was nearly three times that of normal subjects (24.0%).

### 3.2. HRR1 in IPAH Patients and Healthy Subjects

Heart rate recovery was consistently slowed in IPAH patients as compared with healthy subjects after 1 min. Sun et al. report that HRR1 < 16 was highly correlated with several previously published indicators of poor prognosis in IPAH patients [16]. Based on this value, 31 (53.4%) of 58 patients had HRR1 < 16, and 27 (46.6%) of 58 patients had HRR1 ≥ 16.

### 3.3. HRR1 as a Key Abnormal Parameter in IPAH Patients

HRR1 correlated significantly with FVC, FEV1, FEV1% pred, FEV1/FVC (%), FEF50, FEF50%, FEF75, FEF75%, and DLco. HRR1 correlated significantly with NYHA FC, peak VO_2_/kg, and peak VO_2_. IPAH patients with HRR1 <16 had lower peak oxygen pulse compared to the other group. However, there were no statistically significant differences with peak PeTO_2_, peak PeTCO_2_, and VE/VCO_2_ slope between the two groups. Compared to IPAH patients with HRR1 < 16, those with HRR1 ≥ 16 had no significant differences with targeted drug therapy and also had no statistical difference in SBP (systolic blood pressure), DBP (diastolic blood pressure), mPAP, CO, CI, PVR, and PAWP (*P* > 0.05). The mRAP was significantly lower in the patients with HRR1 < 16 compared to those with HRR1 ≥ 16 (Table 2).

### 3.4. Correlates and Predictors of HRR1 in IPAH Patients and Healthy Subjects

Compared with IPAH patients with HRR1 < 16, those with HRR1 ≥ 16 had better NYHA FC scores and better 6-minute walking capacity. In addition, patients with HRR1 ≥ 16 showed less severe abnormalities on metabolic, cardiovascular, and ventilatory responses during CPET. Resting HR was lower, and peak HR tended to be higher in patients with HRR1 ≥ 16; consequently, HR response during exercise was greater in these patients (*P* < 0.05; Table 2).

Consistent with these data, HRR1 ≥ 16 had a higher negative predictive value to rule out severe exercise impairment as indicated by selected maximal and submaximal CPET variables. A number of clinical, hemodynamic, PFT, and CPET-based variables were related to HRR1 as shown in Tables 3 and 4.

## 4. Discussion

The present study highlights the value of chronotropic incompetence and heart rate recovery immediately after cardiopulmonary exercise test as independent predictors of exercise capacity in patients with IPAH. The principal findings of this study were the following: (1) HRR1 was significantly lower in IPAH than that in normal subjects; (2) CRI was detected in 89.7% IPAH patients (*n* = 52), 53% with HRR < 16 beats; (3) a decrease in the heart rate recovery after exercise was associated with worse NYHA functional class, impaired chronotropic response, increased NT-proBNP, and decreased exercise capacity. Although CPET is a noninvasive and inexpensive measure of exercise capacity [11], the test also provides other important caveats. Compared with another commonly used test, the 6-minute walking distance, it is much more objective and can provide more information [11, 12]. Moreover, CPET is more useful for clinical assessment and prognosis evaluation in IPAH patients [14, 17]. CPET parameters like peak VO_2_, OUES, and VE/VCO_2_ have proved to be reliable predictors of disease severity and mortality in PAH patients [5, 6, 10], yet the role of CRI remains undefined. HRR1 is a simple, inexpensive, easily collected variable when the subject performs a CPET and increasingly used in evaluation and follow-up of PAH patients [10, 18].

We also observed slightly decreased PFT parameters in IPAH patients; similar finding has been reported in a previous study as well [19]. Although precise mechanisms leading to airway obstruction in pulmonary hypertension are still unknown, studies by Fernandez-Bonetti and others have suggested peripheral changes in the pulmonary vasculature and abnormal endothelial function may play an important pathophysiological role [20, 21]. It is well known that both sympathetic activation and parasympathetic withdrawal work to increase the HR during exercise [8–10, 22, 23]. After peak exercise, sympathetic withdrawal and parasympathetic activation both contribute towards the recovery of the heart rate [24]. In normal subjects, heart rate is maintained by a sympathovagal balance [25, 26]. In our cohort of HRR1 < 16 patients, the mean HR at baseline was 89.2 ± 12.4 compared to 80.93 ± 10.10 in patients with HRR > 16. AT the same time HR at the end of exercise in HRR < 16 patients were lower than in HRR > 16 patients (135.7 ± 22.2 versus 146.4 ± 19.2), with the HRR1 difference between the two groups being statistically significant (11.0 ± 4.0 versus 23.3 ± 6.8  *P* < 0.01). What is interesting here is that the small airway obstruction along with the DLco was more deranged in HRR < 16 group of patients that is, FEF50, FEF50% FEF75, FEF75%, DLco (% pred) (*P* < 0.05) (Table 3). This higher incidence of obstruction perhaps leads to a more imbalanced sympathovagal tone. We know that airway obstruction causes increased cholinergic (vagal) response.

Recently, Ciarka and colleagues reported autonomic dysfunction and sympathetic overactivity in PAH patients [27]. We think sympathetic overactivity also contributes a lot towards the decrease of HRR1 in IPAH patients. Other studies also indicate that sympathetic overactivity is associated with survival in PAH patients [28]. We also observed an impaired chronotropic response to exercise and a delayed heart rate recovery, which indicates a significant cardiac autonomic abnormality in IPAH patients, corroborating earlier reports [8, 9, 29]. IPAH group had lower CR, ΔHR, and HRR1 than healthy controls. Another interesting finding was, the higher the workload in IPAH patients reached during CPET, the greater the ΔHR; the greater ΔHR, the greater HRR1; this corroborates with the study by Swigris et al. [30]. They assessed heart rate response during 6MWT in subjects with idiopathic pulmonary fibrosis associated pulmonary hypertension. They reported that, with an increase of 1 bpm in ΔHR, peak workload increased by 4.2 W. In our study, in IPAH patients who underwent CPET, with 1 bpm decrease in ΔHR the peak workload decreased by 2.3 W and with 1 bpm increase in HRR1 peak workload increased by 6.1 W. In addition, patients who failed to reach 80% of the age-predicted heart rate were more likely to have a HRR1 < 16 beats. The prevalence of CRI was also higher in such patients.

**Figure 1:**
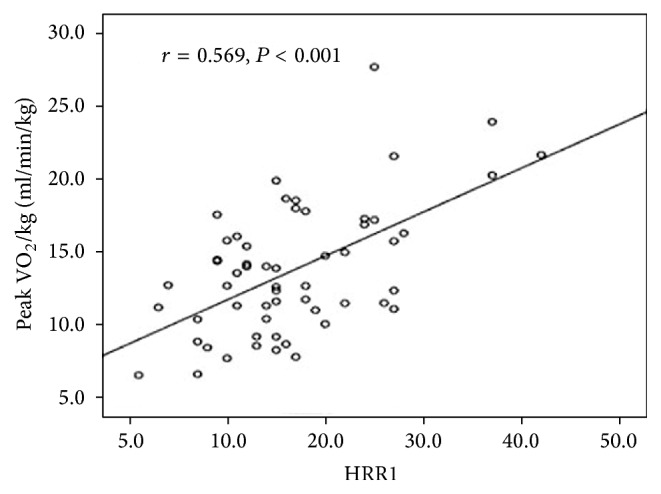
Scatterplot showing the relationship between HRR1 (bpm) and peak VO_2_/kg (ml/min/kg): *r* = 0.569, *P* < 0.001.

HRR1 < 16 beats was associated with severe exercise impairment, higher NYHA function classes, and higher NT-proBNP in the present study (Table 4). Peak VO_2_ is the most widely used parameter to estimate exercise capacity by CPET [6]. Peak VO_2_ was significantly lower in patients with HRR1 < 16 beats and showed a positive correlation with HRR1 (*r* = 0.6, *P* < 0.001, Figure 1), which indicates that HRR1 could also be a predictor of exercise capacity. An increase in right ventricular afterload can lead to an impaired stroke volume (SV) response to exercise. Although an increased afterload does not usually affect the stroke volume in healthy individuals, an unchecked afterload in disease states will affect ventricular ejection thereby affecting the stroke volume. Perhaps an increase in the pulmonary vascular resistance affects the afterload even more in IPAH. Elevated vascular resistance and pulmonary artery pressure can lead to an increasing right ventricular afterload, which could induce sympathetic activity and affect HR during exercise [27, 31].

RHC parameters between HRR1 < 16 and HRR1 > 16 patient groups were not remarkably different (Table 2). The magnitude of change in those parameters with exercise perhaps would be more obvious on a dynamic RHC test. Nevertheless, O_2_-pulse correlated with HRR1 (*r* = 0.4, *P* < 0.01, Table 4) which indicates that HRR1 also reflects a decreased stroke volume in our study. This is based on the concept put forth by Sun et al., who reported that O_2_-pulse can be used as an estimator of stroke volume response to exercise [32]. Unsurprisingly, IPAH patients with HRR1 < 16 had lower peak oxygen pulse compared to those with better heart rate recovery (*P* < 0.05) in the present study. Azarbal et al. evaluated a total of 10021 HF patients and found that CRI was an important predictor of cardiac death and all-cause mortality [33]. Additionally, percent HR reserve was superior to the maximal age-predicted heart rate in predicting cardiac death.

Our study has some limitations. Due to its retrospective design the data collection was not systematically implemented. We did not exclude patients who were on medications like bosentan or sildenafil, which may influence the HR response, but patients who were on beta-blockers were excluded. Analysis of prognosis was difficult as only a few patients died during the study period. Due to the lack of echocardiography data, the relation between HRR and right heart function was not determined. Finally, time frame for performing 6MWD, CPET, and RHC was variable in a few patients.

Despite the limitations, these findings reflect that heart rate recovery is correlated with exercise capacity and the underlying cardiac autonomic abnormalities are significant in IPAH patients. In addition, the result shows that a decrease in HRR1 after CPET was more likely to indicate an impaired exercise capacity and increased disease severity in IPAH patients. Indeed, this study was not designed to prove that HRR could replace peak VO_2_ in evaluating exercise capacity. The goal was to help us understand autonomic dysfunction in IPAH patients and identify a new variable that can be helpful in assessing disease severity. Further studies are warranted to investigate the role of HRR and CRI in evaluation of prognosis and efficacy of drugs and to find out the mechanisms that can further explain the change of HR associated variables during CPET.

## 5. Conclusions

In summary, a decrease in HRR1 in IPAH patients is associated with severe limitation of exercise capacity. HRR1 < 16 beats and CRI just after completion of a CPET could be an indicator of poor prognosis. We believe HRR1 and CR could be used as an outcome measure and measure of improvement in exercise capacity and in trial of therapy for IPAH.

## Figures and Tables

**Table 1:** The demographics, PFT, and CPET of IPAH variables in patients and healthy subjects.

	IPAH patients	Control subjects	*P*
Demographics			
*N*	58	25	
Sex (F/M)	37/21	13/12	>0.05
Age (y)	35.2 ± 13.8	40.1 ± 15.4	>0.05
BMI (kg/m^2^)	22.5 ± 4.2	22.3 ± 3.4	>0.05
PFT			
FVC (L)	2.9 ± 0.9	3.7 ± 0.9	<0.01
FVC% pred	81.9 ± 15.4	97.2 ± 13.1	<0.01
FEV_1_ (L)	2.4 ± 0.7	3.0 ± 0.7^*∗*^	<0.01
FEV_1_% pred	77.4 ± 14.8	96.2 ± 11.5	<0.01
FEV_1_/FVC (%)	80.2 ± 7.5	83.2 ± 6.7^*∗*^	>0.05
FEF25 (L/s)	4.7 ± 2.0	6.0 ± 1.8	<0.05
FEF25%	72.5 ± 22.0	94.6 ± 11.4	<0.01
FEF50 (L/s)	2.8 ± 1.4	4.2 ± 1.3^*∗*^	<0.01
FEF50%	66.0 ± 25.7	102.3 ± 29.5^*∗*^	<0.01
FEF75 (L/s)	1.0 ± 0.8	2.0 ± 1.8^*∗*^	<0.05
FEF75%	49.8 ± 27.9	89.5 ± 32.7^*∗*^	<0.01
DLco (% pred)	65.1 ± 11.3	92.3 ± 11.0	<0.01
CPET			
Base line HR (bpm)	85.4 ± 12.0	84.0 ± 10.7	>0.05
Peak HR (bpm)	140.7 ± 21.3	167.4 ± 17.1	<0.01
ΔHR (bpm)	55.3 ± 21.3	83.4 ± 14.5	<0.01
HR 1 min post (bpm)	123.9 ± 20.7	139.6 ± 20.1	<0.01
HRR1 (bpm)	16.8 ± 8.2	27.8 ± 7.1^*∗∗*^	<0.01
HRR1 (<16 bpm), *N*	31 (53.4%)	6 (24.0%)	<0.01
Peak exercise HR < 80% of predicted, *N* (%)	52 (89.6%)	2 (8.0%)	<0.01
CR	0.6 ± 0.2	0.9 ± 0.1	<0.01
CRI, *N* (%)	52 (89.7%)	6 (24.0%)	<0.01
Peak Load (W)	65.1 ± 26.4	144.0 ± 45.4^*∗∗*^	<0.01
Peak VO_2_ (ml/min)	779.9 ± 265.2	1659.9 ± 381.8^*∗∗*^	<0.01
Peak VO_2_% pred	41.1 ± 11.8	83.9 ± 13.4^*∗∗*^	<0.01
Peak V_E_ (L/min)	43.64 ± 16.3	63.1 ± 14.7^*∗*^	<0.01
Peak BF (breaths/min)	33.8 ± 7.1	35.5 ± 6.6	>0.05
Peak O_2_ pulse (ml/beat)	5.6 ± 1.9	10.1 ± 2.0^*∗*^	<0.01
VE/VCO_2_ slope	41.5 ± 10.3	28.63 ± 3.9	<0.01

^*∗*^*P* < 0.05, ^*∗∗*^*P* < 0.01, versus controls using unpaired *t*-test.

Values are presented as means ± SD. IPAH = idiopathic pulmonary arterial hypertension; % pred = percent of predicted; *N* = number; BMI = body mass index; PaO_2_ = partial arterial oxygen pressure; PaCO_2_ = partial arterial carbon dioxide pressure; PFT = pulmonary function test; FVC = forced vital capacity; FEV_1_ = forced expiratory volume in 1 second; FEF25 = forced expiratory flow at 25% of forced vital capacity; FEF50 = forced expiratory flow at 50% of forced vital capacity; FEF75 = forced expiratory flow at 75% of forced vital capacity; DLco = gas transfer index or diffusing capacity for carbon monoxide; CPET = cardiopulmonary exercise test; Peak VO_2_ = peak oxygen uptake; VE = minute ventilation; VCO_2_ = carbon dioxide output; BF = breath frequency; HR = heart rate; ΔHR = heart rate changes from rest to peak; HRR1 = heart rate recovery at 1 minute; CR = chronotropic reserve; CRI = chronotropic incompetence.

**Table 2:** Summary of demographics, PFT, and CPET of IPAH patients with HRR1 < 16 and HRR1 ≥ 16.

	IPAH patients with HRR1 < 16	IPAH patients with HRR1 ≥ 16	*P*
Demographics			
*n*	31	27	
Sex (F/M)	22/9	15/12	>0.05
Age (y)	37.7 ± 13.4	32.7 ± 14.1	>0.05
BMI (kg/m^2^)	22.4 ± 3.3	22.6 ± 5.2	>0.05
PFT			
FVC (L)	2.8 ± 0.9	3.2 ± 0.9	>0.05
FVC% pred	80.2 ± 16.1	84.0 ± 14.5	>0.05
FEV_1_ (L)	2.2 ± 0.7	2.6 ± 0.7^*∗*^	<0.05
FEV_1_% pred	74.6 ± 13.8	80.8 ± 15.7	>0.05
FEV_1_/FVC (%)	78.1 ± 7.9	82.8 ± 6.0^*∗*^	<0.05
FEF25 (L/s)	4.5 ± 2.1	5.1 ± 1.9	>0.05
FEF25%	70.4 ± 24.5	75.8 ± 17.9	>0.05
FEF50 (L/s)	2.4 ± 1.2	3.4 ± 1.4^*∗*^	<0.05
FEF50%	59.6 ± 24.7	75.9 ± 24.7^*∗*^	<0.05
FEF75 (L/s)	0.8 ± 0.6	1.4 ± 1.1^*∗*^	<0.05
FEF75%	41.6 ± 24.0	62.3 ± 29.6^*∗*^	<0.05
DLco (% pred)	61.1 ± 12.3	70.5 ± 16.3^*∗*^	<0.05
Clinical parameters			
NYHA FC			<0.05
1,2, *n* (%)	11 (35.5%)	22 (81.5%)	
3,4, *n* (%)	20 (64.5%)	5 (18.5%)	
NT-proBNP (pg/ml)	1354.7 ± 1613.4	365.4 ± 358.4^*∗∗*^	<0.01
6MWD (m)	443.8 ± 84.0	489.3 ± 69.3^*∗*^	<0.05
Background therapy			>0.05
Sildenafil, *n*	8	9	
Tadalafil, *n*	3	3	
Vardenafil, *n*	4	5	
Bosentan, *n*	8	6	
Ambrisentan, *n*	4	2	
Ventavis, *n*	4	2	
RHC			
SBP, mmHg	119.0 ± 18.0	109.5 ± 16.9	>0.05
DBP, mmHg	74.6 ± 13.2	72.1 ± 14.6	>0.05
mPAP, mmHg	58.8 ± 13.2	59.0 ± 15.4	>0.05
mRAP, mmHg	6.5 ± 4.1	9.2 ± 4.7	<0.05
CO, l/min	4.5 ± 1.2	4.9 ± 2.0	>0.05
CRI, l/min/m2	2.9 ± 0.8	2.9 ± 1.0	>0.05
PVR, mmHg	12.5 ± 5.8	12.8 ± 7.0	>0.05
PAWP, mmHg	7.9 ± 3.5	8.4 ± 3.8	>0.05
CPET			
HRR1 (bpm)	10.9 ± 3.8	23.7 ± 6.8^*∗∗*^	<0.01
HR at baseline (bpm)	89.2 ± 12.4	80.9 ± 10.1	<0.01
HR at end (bpm)	135.7 ± 22.2	146.4 ± 19.2	>0.05
ΔHR(bpm)	46.4 ± 19.3	65.5 ± 19.2	<0.01
HR 1 min post (bpm)	124.6 ± 21.7	123.1 ± 19.9	>0.05
HRR1 (bpm)	11.0 ± 4.0	23.3 ± 6.8	<0.01
CR	0.5 ± 0.29	0.6 ± 0.2	<0.01
CRI (*N*)	28 (90.3%)	24 (88.9%)	>0.05
Peak Load (W)	56.3 ± 22.8	75.6 ± 26.9^*∗∗*^	<0.01
Peak VO_2_ (ml/min)	689.4 ± 210.8	891.2 ± 286.2^*∗∗*^	<0.01
Peak VO_2_% pred	37.2 ± 11.2	45.9 ± 11.0^*∗∗*^	<0.01
Peak HR (beats/min)	136.5 ± 22.5	145.1 ± 18.7	>0.05
Peak V_E_ (L/min)	42.3 ± 18.2	45.3 ± 13.7^*∗*^	<0.05
Peak BF (breaths/min)	34.1 ± 7.4	33.5 ± 6.9	>0.05
Peak O_2_ pulse (ml/beat)	5.2 ± 1.7	6.20 ± 1.9^*∗*^	<0.05
Peak PET CO_2_	23.5 ± 9.4	25.7 ± 6.6	>0.05
Peak PET O_2_	127.4 ± 9.7	124.9 ± 6.5	>0.05
VE/VCO_2_ Slope	42.0 ± 12.0	41.0 ± 8.3	>0.05

Values are expressed as mean ± SD.

^*∗*^*P* < 0.05, ^*∗∗*^*P* < 0.01, versus IPAH patients with HRR1 ≥ 16 using unpaired *t*-test.

NT-proBNP = n-terminal natriuretic peptide type-B; FC = New York Heart Association functional classification; 6MWD = 6-minute walking distance; PETCO_2_ = partial pressure of end-tidal carbon dioxide; PETO_2_ = partial pressure of end-tidal oxygen; SBP = systolic blood pressure; DBP = diastolic blood pressure; other abbreviation definitions are same as Table 1.

**Table 3:** The correlation between PFT parameters and HRR1 of IPAH patients.

Parameters	*r*	*P*
FVC (L)	0.3	<0.05
FEV1 (L)	0.4	<0.01
FEV_1_% pred	0.3	<0.05
FEV1/FVC (%)	0.4	<0.05
FEF50 (L/s)	0.5	<0.01
FEF50%	0.5	<0.01
FEF75 (L/s)	0.5	<0.01
FEF75%	0.5	<0.01
DLCO	0.3	<0.05

The abbreviation definitions are same as Table 1.

**Table 4:** The correlation between demographics, 6MWD, CPET parameters, and HRR1 of IPAH patients.

Parameters	*r*	*P*
NYHA FC	−0.6	<0.01
NT-proBNP (pg/ml)	−0.3	<0.01
6MWD (m)	0.4	<0.01
Peak Load (W)	0.5	<0.01
peak VO_2_/kg (ml/min/kg)	0.56	<0.01
Peak VO_2_ (ml/min)	0.6	<0.01
Peak VO_2_% pred	0.5	<0.01
Peak O_2_ pulse (ml/beat)	0.4	<0.01

The abbreviation definitions are same as Tables 1 and 2.
